# Zebra Finch Song Phonology and Syntactical Structure across Populations and Continents—A Computational Comparison

**DOI:** 10.3389/fpsyg.2016.00980

**Published:** 2016-07-07

**Authors:** Robert F. Lachlan, Caroline A. A. van Heijningen, Sita M. ter Haar, Carel ten Cate

**Affiliations:** ^1^Behavioural Biology, Faculty of Science, Institute of Biology, Leiden UniversityLeiden, Netherlands; ^2^Department of Psychology, School of Biological and Chemical Sciences, Queen Mary University of LondonLondon, UK; ^3^Leiden Institute for Brain and Cognition, Leiden UniversityLeiden, Netherlands; ^4^Faculty of Humanities, Leiden University Centre for Linguistics, Leiden UniversityLeiden, Netherlands

**Keywords:** birdsong, cultural evolution, vocal communication, computational acoustics, zebra finch

## Abstract

Learned bird songs are often characterized by a high degree of variation between individuals and sometimes between populations, while at the same time maintaining species specificity. The evolution of such songs depends on the balance between plasticity and constraints. Captive populations provide an opportunity to examine signal variation and differentiation in detail, so we analyzed adult male zebra finch (*Taeniopygia guttata*) songs recorded from 13 populations across the world, including one sample of songs from wild-caught males in their native Australia. Cluster analysis suggested some, albeit limited, evidence that zebra finch song units belonged to universal, species-wide categories, linked to restrictions in vocal production and non-song parts of the vocal repertoire. Across populations, songs also showed some syntactical structure, although any song unit could be placed anywhere within the song. On the other hand, there was a statistically significant differentiation between populations, but the effect size was very small, and its communicative significance dubious. Our results suggest that variation in zebra finch songs within a population is largely determined by species-wide constraints rather than population-specific features. Although captive zebra finch populations have been sufficiently isolated to allow them to genetically diverge, there does not appear to have been any divergence in the genetically determined constraints that underlie song learning. Perhaps more surprising is the lack of locally diverged cultural traditions. Zebra finches serve as an example of a system where frequent learning errors may rapidly create within-population diversity, within broad phonological and syntactical constraints, and prevent the formation of long-term cultural traditions that allow populations to diverge.

## Introduction

The phenotypic diversity of an animal signal reflects the way in which it develops. Learned bird song develops from an interplay of imitative vocal learning and unlearned genetically based biases and predispositions (Catchpole and Slater, 2008). The relatively high error rates inherent to cultural transmission typically result in correspondingly high levels of within-population diversity (Podos and Warren, 2007). This diversity may be constrained, however, either by morphological constraints on the sounds birds can produce (Podos et al., 1999; Tierney et al., 2011), or perceptual predispositions for the sounds perceived as belonging to conspecifics (Peters et al., 1980; Nelson, 2000). At the extreme it has been proposed that, in some species, like swamp sparrows (*Melospiza georgiana*), song learning may be restricted to selecting the building blocks of their songs from a genetically encoded list of syllable type categories (Marler and Pickert, 1984; Marler, 1997).

While it is clear that in songbirds both cultural transmission and genetic constraints play a role in development, their relative contributions to population differentiation remain generally unclear (Podos and Warren, 2007). The rapid tempo of cultural evolution means that novel song forms frequently arise and can quickly become established in a population. This can promote population divergence (especially in “dialect” forming species, e.g., Baptista, 1977; Podos et al., 2004). Population divergence in song characteristics requires that novel song types (through learning errors or innovation) are generated frequently enough to overwhelm the homogenizing effects of the dispersal of individuals and songs between populations (e.g., when songs from one population are learned by those from another). On the other hand, however, if learning errors occur too frequently and are too large in magnitude, then any culturally transmitted population-specific signatures will be lost. Within-population variation in song characteristics will be very high, and will only be constrained by underlying experience-independent predispositions for song learning. For populations that have not yet diverged in the genes underlying these predispositions, this means that song characteristics will largely overlap between populations.

**Figure 1:**
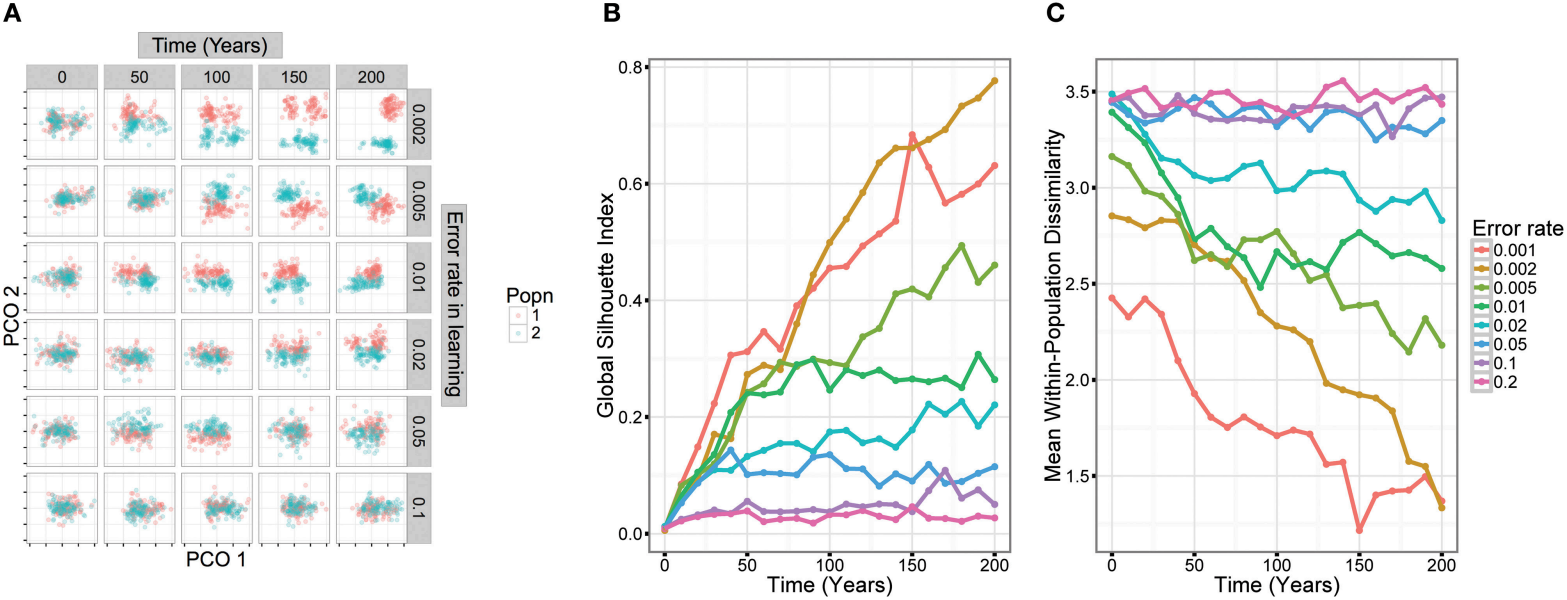
**Simulation models demonstrate how population divergence depends on low error rates in song learning**. In the models, two small populations were founded from a larger population. Songs were simulated in a way that approximated zebra finch song structure and culturally evolved over a period of 200 years. We then examined divergence in song structure over this period. **(A)** The trajectory of song cultural evolution in two populations in five runs of the simulation with different error rates. Each graph represents a song as a point located on two dimensions calculated by principal coordinates analysis (each tick mark represents one unit in the PCO space). An error rate of 0.1 means that there was a 90% chance that any particular syllable was learned precisely from another male in the population, and a 10% chance that it was improvised, taking a random value from within the species-specific range. **(B)** Divergence in song structure over time. We used the Global Silhouette Index to quantify song divergence between the two populations—higher values suggest that the two clusters are well-separated. **(C)** Within-population diversity is low when error rates in learning are low. But as error rates increase above 0.05, diversity approaches a ceiling. This reflects the fact that the population covers the entire range of species-specific syllable structures.

To verify this intuition about the limits of cultural divergence, we carried out simple simulations of cultural evolution using different error rates in learning (Figure 1), and with parameters that broadly approximated zebra finch song (see Appendix in Supplementary Material for methodological details). These simulations, clearly demonstrated that as error rates increased, population divergence decreased. At error rates greater than ~0.05, there was little clear divergence between populations.

Studies of song evolution have focused in particular on the processes of divergence occurring in the period just after the founding of new populations—especially on islands. Such events are often associated with a large loss of song diversity—presumably the consequence of cultural bottlenecks, as only a small number of individuals and a limited set of songs will be present in the founding population (e.g., Baker, 1996; Lachlan and Slater, 2003; Parker et al., 2012). In other examples, founding of new populations appears to lead to rapid song divergence from the original population (Baker et al., 2006; Lachlan et al., 2013). One possible explanation for this is that recently established populations consist of a large proportion of naïve individuals who improvise songs in the absence of sufficient tutors, similar to birds raised in isolation in the laboratory (Thielcke, 1973), and establish new cultural traditions in the population. But if error rates in song learning are high enough, neither of these processes of cultural evolution would be sufficient to allow population differentiation. Instead, one would expect that songs within the population should re-converge on pre-colonization norms within several generations (Fehér et al., 2009).

Zebra finches have been a model species for song learning and other behavioral studies since the 1960's, and as a result are bred in many research colonies. These domesticated populations typically derive from animals captured in their native Australia within the last 150 years (Sossinka, 1970; Rogers, 1979). They therefore provide an opportunity to explore how song can diverge in isolated populations: while some colonies are replenished from breeders, other laboratories have their own breeding program. In spite of occasional interchange of animals between laboratories, populations show significant genetic differentiation (Forstmeier et al., 2007), suggesting at least some degree of isolation. Each male zebra finch precisely imitates some of the elements that make up its song (Tchernichovski and Mitra, 2002), but it is equally clear that errors in learning are common: it is fairly uncommon for all elements within a zebra finch song to be copied, while it is normal that a pupil's song may contain some novel elements. In one aviary learning study, an average of 1.24 elements per song (12% of the elements within the song) could not be assigned to any tutor (Mann and Slater, 1995), whereas several studies on laboratory populations of zebra finches show even higher error rates of ~50% (Jones et al., 1996; Houx and ten Cate, 1999; Holveck et al., 2008). Despite this relatively high error rate, there are preliminary indications that different laboratory populations might develop different vocal traditions and differ in song features (Sturdy et al., 1999). This raises the question of how readily populations diverge in their song and in which way. In a detailed study, Zann (1993b) examined divergence in song structure among wild populations of zebra finches. While he found divergence in some song features at a continent-wide scale, there was little divergence between local populations. Zann concluded that frequent dispersal between populations prevents local song divergence. An alternative hypothesis, however, is that imprecise song imitation prevents local song divergence. Laboratory populations of zebra finches offer an opportunity to test these hypotheses. Based on our simulations (Figure 1), and an estimated error rate in zebra finch syllable learning of >10% (12–50%, see above), we would expect cultural song divergence to be limited even when genetic markers have diverged.

Zebra finches also provide an opportunity to investigate how domestication influences song evolution. Domestication may be similar in many respects to island colonization, involving population bottlenecks and relaxation of sexual selection. In line with this, and mirroring Thielcke's theory about song evolution after island colonization, the song structure of the Bengalese finch (a member of the estrildidae, like the zebra finch) diverged rapidly from the original population's song following domestication (Honda and Okanoya, 1999). In a recent study of zebra finches, however, colonization was simulated in a laboratory setting by using naïve non-tutored individuals as tutors for the next generation. Over subsequent tutoring generations, songs converged onto species-typical norms within only a few generations, suggesting that song learning errors in combination with learning or production biases are common enough to prevent population divergence (Fehér et al., 2009).

**Figure 2:**
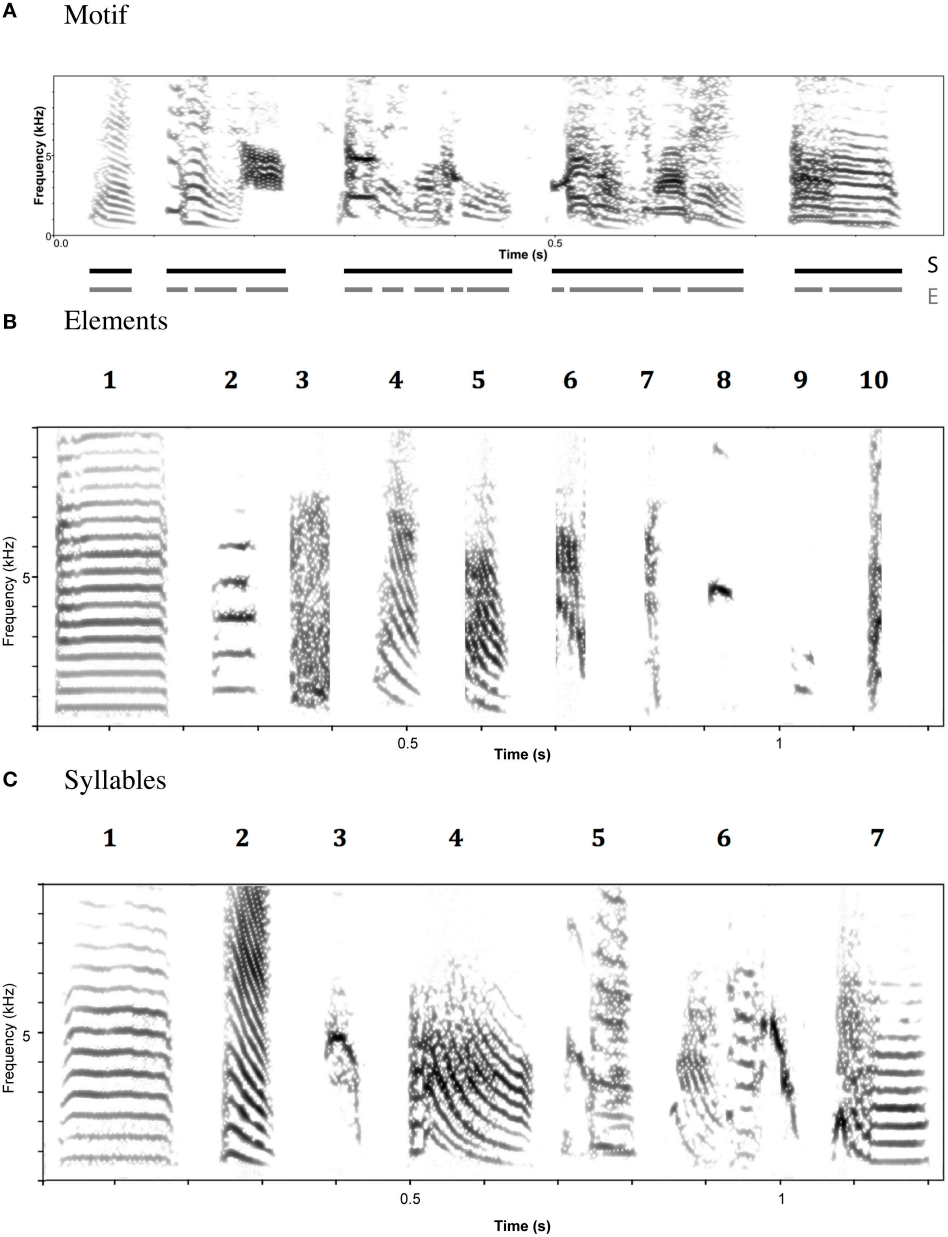
**Example spectrograms of zebra finch song (A), element categories (B), and syllable categories (C)**. Element and Syllable category labels refer to descriptions in the text.

The first aim of this study is to characterize the species-wide structure of zebra finch songs. By “structure” we mean the acoustic features of the component units of the song and the way in which these units are sequenced within a song. Zebra finch songs (defined as a period of uninterrupted singing) consist of repeated, more or less stereotyped strings (“motifs”) of different sound units (“syllables” and “elements”; Figure 2). Unlike some other species, elements are rarely immediately repeated within the motif. An initial challenge is to segment motifs into their constituent units. At the most fine-grained level, motifs might be divided into individual vocal gestures. Here, we call such units “elements.” Element boundaries can only be inferred indirectly, but it is possible to discern rapid changes in acoustic parameters—for example in the slope of fundamental frequency—that seem likely to correspond to boundaries between elements (ten Cate et al., 2013).

There is also evidence for units at a higher hierarchical level within motifs. These units, called here “syllables” can be characterized by the presence or absence of inter-element gaps. Within syllables, there are very short, or no gaps between elements; between syllables, there are larger gaps. Evidence from development and song production studies suggest that syllables are biologically meaningful (ten Cate et al., 2013). In our study we analyzed songs at both the element and syllable levels.

We tested the degree to which elements or syllables could be grouped into species-wide clusters, the degree to which element or syllable categories showed syntactical dependencies, and the degree to which zebra finch populations differed from one another in the acoustic structure of elements or syllables. To do this, we harnessed a recently developed method for the computational comparison of complex vocal signals (Lachlan et al., 2010, 2013) to cluster analysis techniques, and examined intra-population and species-wide (i.e., inter-population) patterns of song variation in captive and wild populations of zebra finches.

## Materials and methods

We assembled recordings from 12 laboratory populations and one wild population of zebra finches. The 12 laboratory populations were spread out across research laboratories in Europe, America and New Zealand (see Table 1 for an overview of the various colonies and the sample sizes). Most were derived from breeders' stock (and thus were likely derived from wild stock in the nineteenth century). The wild zebra finch recordings were made in the field or shortly after capture (in a field aviary) in two separate locations (Alice Springs, Central Australia, and Northern Victoria). Songs were recorded in host laboratories using different recording equipment. Zebra finch songs consist of “motifs”—more or less stereotyped sequences of elements that are sung in succession, preceded by some “introductory elements.” We selected one exemplar song with good recording quality for each individual. From this song, we selected the predominant motif (the one with the most common element sequence) for analysis. Some songs were recorded in female-directed context others were undirected. Undirected song might show slightly higher within-individual diversity than directed song, but current knowledge (Woolley and Doupe, 2008) suggests even within-individual differences are very subtle and hence we don't expect this to influence our results about element and syllable repertoires or population differences. Introductory notes were excluded unless they recurred in motifs later during the song, because introductory notes would potentially bias the automatic acoustic analysis and probably have very little variation between individuals. Motifs were selected by two observers (StH and CvH). Each observer selected half of the motifs for each population, reducing the possibility of an observer bias. Songs that were not recorded at a 22.05 kHz sampling rate were resampled to that rate.

**Table 1:** **Details of zebra finch colonies sampled for this study**.

**Name**	**Researcher providing recordings**	**Institute**	**Sample size (males)**	**Directed or undirected song**
Auckland	M. E. Hauber and D. L. M. Campbell	University of Auckland, Auckland, New Zealand	9	Directed
Bielefeld	M. Honarmand and M. Naguib	Universität Bielefeld, Bielefeld, Germany	18	Directed
Berlin	C. Scharff and J. Rautenkranz	Freie Universität Berlin, Berlin, Germany	15	Undirected
Columbia	S. Woolley and A. Vyas	Columbia University, New York, NY, USA	14	Directed
Hunter	C. Harding and A. Vyas	Hunter College, New York, NY, USA	15	Directed
Leiden	M. Holveck	Leiden University, Netherlands	15	Undirected
LaTrobe	R. A. Zann	La Trobe University, Melbourne, Australia^*^	17	Both
Montreal	N. J. Boogert	McGill University, Montreal, Canada	15	Directed
St. Andrews	H. Brumm	University if St. Andrews, UK	14	Directed
St. Etienne	C. Vignal	University of Saint-Etienne, France	15	Directed
San Francisco	A. J. Doupe	University of California San Francisco, CA, USA	15	Undirected
Seewiesen	A. Leitão	Max Planck Institute for Ornithology Seewiesen, Germany	13	Undirected
Williams College	H. Williams	Williams College, Williamstown, MA, USA	14	Directed

* Based on recordings from wild-caught individuals (see text for more details).

Motifs were analyzed using Luscinia software (http://rflachlan.github.io/Luscinia). Segmentation of motifs into elements was carried out based on visual inspection of the spectrograms by three experienced experimenters (RFL, CvH, StH) to reduce observer bias. Criteria for segmentation were silent gaps (≥0.5 ms) or the presence of abrupt changes in frequency and amplitude in a continuous sound. Segmentation of motifs into syllables was based on the duration of inter-element gaps: gaps >5 ms were taken to signify gaps between syllables.

Our justification for this approach comes partly from studies of other songbirds where syllabic structure is clearer. In swamp sparrows (*M. georgiana*) and chaffinches (*Fringilla coelebs*), among other species, syllables are typically repeated a number of times within the song. The repetition of units therefore provides an alternative basis for segmentation. In these two species, inter-element gaps form a bimodal distribution, with shorter gaps occurring within syllables, and longer gaps occurring between syllables (Lachlan, unpublished data).

**Figure 3:**
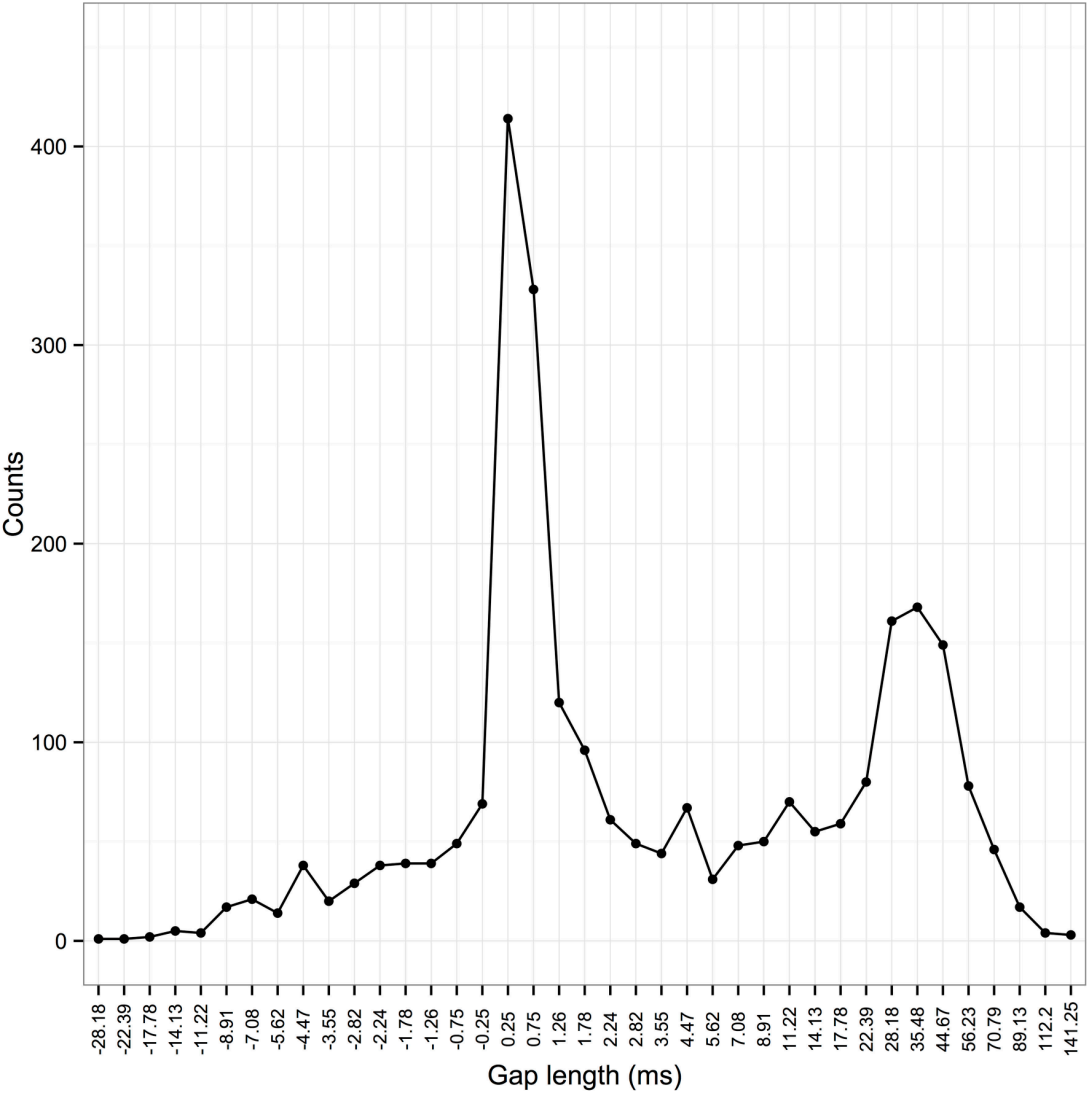
**Frequency distribution of inter-element gaps in zebra finch songs**. The x-axis shows the length of inter-element gaps. Since zebra finch elements can overlap in time, negative gaps are possible [when start time element 2 (st2) < end time element 1 (et1), st2 −et1 is negative]. Elements are grouped into bins with an approximately logarithmic scale (direction reversed for values < 0; bin sizes of 0.5 ms between −1 and 1 ms due to limits in measurements from the spectrogram). Temporal features of acoustic signals appear to be perceived on a logarithmic scale by birds (Dooling and Haskell, 1978).

We therefore analyzed the distribution of inter-element gaps in zebra finches. As with swamp sparrows and chaffinches, these formed a bimodal distribution (Figure 3). Based on this, we set a threshold of 5 ms, above which the elements flanking the gap were placed into different syllables.

A high-pass filter was applied with a default cut-off at 420 Hz. Spectrogram settings were standardized for all songs (256 pts window, 0.5 ms time-step, Hamming window, 100% deverberation factor with a 100 ms window), except for dynamic range, which was varied according to differences in recordings between populations. Luscinia measured contours for a number of acoustic parameters throughout each element. In this study, we used fundamental frequency, fundamental frequency change, mean frequency, and harmonicity (the proportion of energy in the signal's spectrum lying close to an integer multiple of the fundamental frequency). Fundamental frequency (FF) estimation is not straightforward for spectrally complex and noisy sounds like zebra finch elements; in Luscinia, two further parameters (jump suppression and bias) were varied on an element-by-element basis to tune estimates. The FF bias can change the weighting of different hypothesis the program used to calculate fundamental frequency. Correct fundamental frequency estimates were verified by inspection of spectrograms. Frequency change is defined as the slope of the spectrogram at that point. It is expressed on a scale where 0 means decreasing in frequency infinitely quickly, while 1 means increasing in frequency infinitely quickly and 0.5 means not changing in frequency over time. It is calculated by carrying out a linear regression of the fundamental frequency estimate and applying an arctan transformation to the slope. Mean frequency is calculated by summing for each frequency band the frequency multiplied by the spectrogram intensity and dividing the total by the sum of the intensities for the signal. The harmonicity is calculated as the proportion of the intensity of the spectrum that is found at frequencies ±1/4 FF of each harmonic.

A key point is that for each of these parameters, a measurement was made at each time point in the spectrogram, creating a “contour” for how that parameter varied throughout the element. We then compared elements by comparing these contours, preserving all the information about how elements varied over time.

Elements and syllables were compared using an implementation of the dynamic time warping (DTW) algorithm, which has previously been described and successfully applied to zebra finch song (Lachlan et al., 2010). In short, the dynamic time warping algorithm searches for an optimal alignment from one time series to another, allowing the comparison of the contours measured in Luscinia. The algorithm first calculates a dissimilarity matrix between each point in one series and each point in the other. In our case, this was calculated as the Euclidean distance across the normalized five features we used. The algorithm then searches for the optimal path to traverse this matrix from the start to the end of the element/syllable, and then calculates an average of the dissimilarities along this path. A more detailed description of the algorithm and the details of its implementation in Luscinia can be found on https://github.com/rflachlan/Luscinia/wiki/Time-Warping-Analysis. In zebra finches, previous versions of this algorithm have been shown to produce measures of song similarity that are highly correlated with human judgments (Holveck et al., 2008; Lachlan et al., 2010). In this analysis, the four acoustic parameters were weighted by their overall standard deviation. The final, and fifth feature in the DTW was time (i.e., the position of each time point within the element/syllable). This was weighted by *q.p*, where *p* is the inverse length of the longer of the two elements/syllables compared *i* or *j*, and *q* is a weighting parameter that we set to 5 on the basis of inspection of results for the comparison of subsets of the data (See also Lachlan et al., 2013). This normalization ensured a logarithmic like weighting of overall differences in length: all else being equal, 2 ms notes were as different from 4 ms notes as 20 ms notes were from 40 ms notes. These settings assume that all five parameters contribute approximately equally to the overall difference between elements or syllables. The DTW output consisted of a triangular dissimilarity matrix between each pair of elements or syllables in the 13 populations. This formed the basis for further analysis.

We clustered song units using a k-medoids “Partioning Around Medoids” (PAM) clustering analysis (Kaufman and Rousseeuw, 1987), applying the corrected global silhouette index (GSI, Rousseeuw, 1987; Handl et al., 2005) to determine the correct number of clusters. A clear peak in the GSI (range −1 to 1) indicates clustering tendency. We verified our analysis by using an entirely different clustering method, Bayesian Gaussian Mixture models (BGM, using package Mclust in R; Fraley and Raftery, 2006), followed by a merging Gaussians method (method “demp” in function “mergenormals” in package fpc in R, Hennig, 2013). While the Gaussian Mixture Models allowed a more flexible clustering approach (incorporating clusters of different sizes and shapes, for example), it required us to ordinate our DTW dissimilarity matrix into Euclidean space, which we did using Non-metric multidimensional scaling (NMDS, Kruskal, 1964).

To determine the frequency of occurrence of element and syllable types within- and between-populations, we used a permutation Chi-square test. Individuals were permutated 10,000 times randomly between populations to generate distributions of the randomly expected frequency of occurrence of each element or syllable type per population and this number was compared to the observed frequency of occurrence.

To test the magnitude of the divergence between populations, we used a MRPP (multiresponse permutation procedure) approach (MRPP, McCune and Grace, 2002). Individuals were permuted randomly 10,000 times between populations. MRPP compares the observed within-population dissimilarities in element or syllable structure in the original data-set (δ) with those in the permuted data-set (m_δ_), with the expectation that if the former is smaller than the latter, populations have significantly diverged.

We visualized population differentiation in a neighbor-joining dendrogram, where the length of the branches corresponds to the dissimilarity of the population spatial medians. The spatial median within a population were calculated based on NMDS scaling in Euclidean space (as described above for the clustering).

## Results

### Cluster analysis

**Figure 4:**
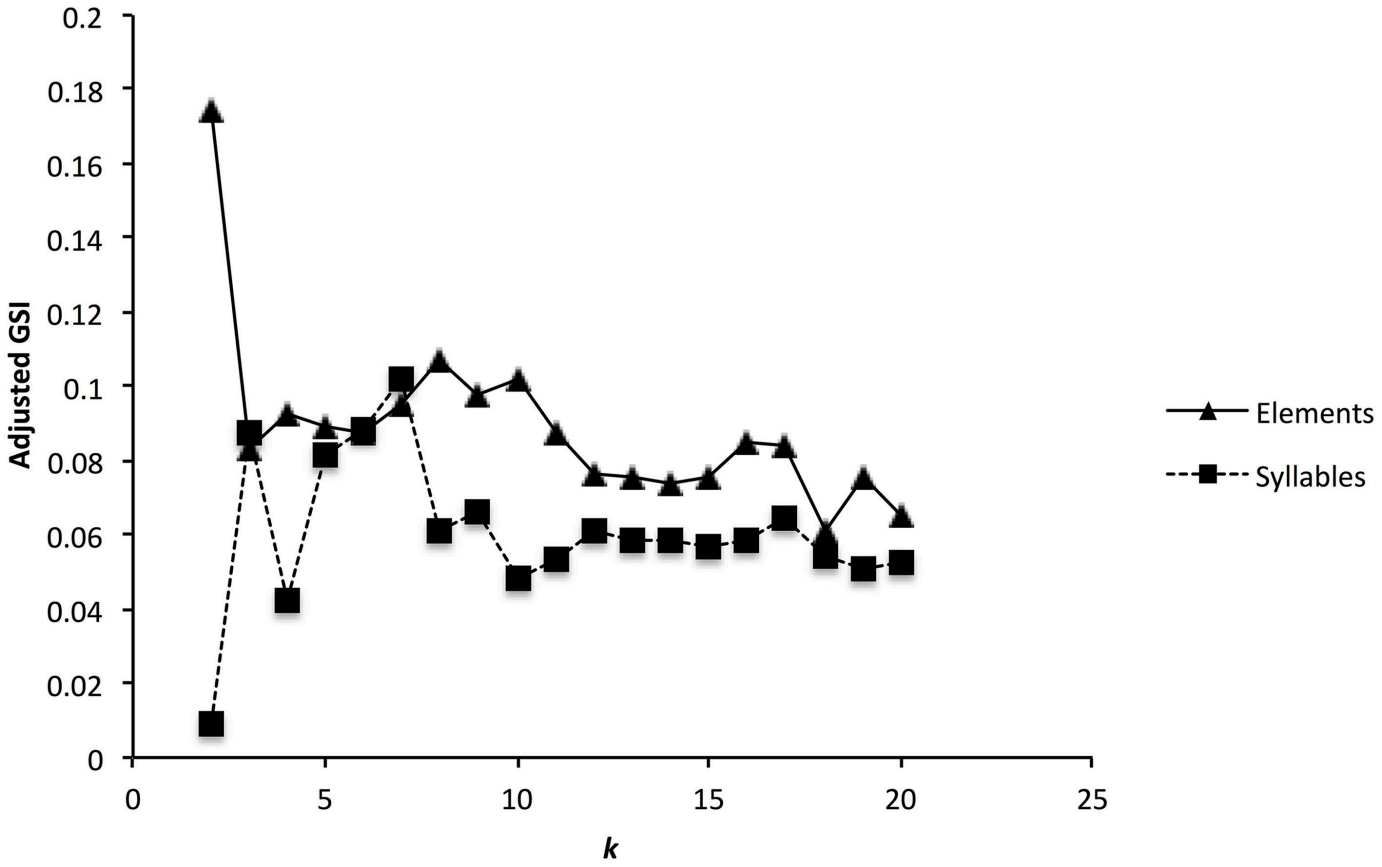
**Cluster validation for zebra finch elements (a) and syllables (b)**. Graph shows the adjusted global silhouette index (GSI) for up to 20 different clusters. Peaks in the GSI correspond to clustering solutions with higher support.

**Figure 5:**
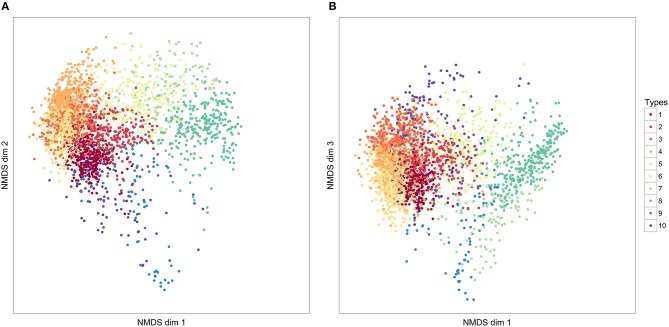
**The distribution of 10 element clusters across three NMDS dimensions**. Each point represents an individual element. The closer elements are to each other in these dimensions, the more similar they have been judged in similarity, according to our DTW comparison of elements. The values in NMDS dimensions are not meaningful, and following convention, axes have not been labeled. **(A)** NMDS dimensions 1 and 2; **(B)** NMDS dimensions 2 and 3.

For elements, the corrected global silhouette index (Rousseeuw, 1987) of the PAM cluster analysis (Kaufman and Rousseeuw, 1987) showed one clear peak, with 2 clusters, which divided high fundamental frequency elements from all other elements (Figure 4). The mean (s.d.) fundamental frequency for elements in cluster 1 was 839 Hz (410 Hz), and for cluster 2 was 3559 Hz (1674 Hz). Each of these two clusters clearly encompassed considerable structural diversity. This diversity did not fall into very clearly defined categories, although there was weak evidence for clustering tendency and we set the threshold for the distinction of clusters at *k* = 10 for further analysis (this value coincided with a weak peak in silhouette index, but should be considered an arbitrary choice). Examples of these 10 clusters are shown in Figure 2B (for descriptions see Table 2, for summary acoustic parameters see Table 4, for an illustration of how these clusters differ in the first three dimensions of a NMDS ordination of the DTW dissimilarities, see Figure 5).

**Table 2:** **Description of element types identified by cluster analysis**.

**Type**	**Description**
1	Long flat notes.
2	High flat notes—higher frequency and often shorter than (1).
3	Noisy, mostly flat notes—noisier than (1,2).
4	Slide notes.
5	Lower slide notes—lower mean and fundamental frequency, higher harmonicity than (4).
6	Noisy, medium high frequency notes—typically declining in fundamental frequency.
7	Declining medium high frequency notes—rapidly declining in frequency, high harmonicity.
8	High frequency notes—higher frequency, harmonicity than (6).
9	Low notes—low fundamental frequency, low mean frequency, high harmonicity notes; typically flat.
10	Upsweep notes.

These results were broadly supported by a Bayesian Gaussian Mixture models (BGM, MClust/demp in R; Fraley and Raftery, 2006; Hennig, 2013) analysis of 3 NMDS dimensions of the element dissimilarity matrix (which explained 87.9% of the total variation between elements). First, the optimal clustering model was selected (using the MClustBIC function in R, selecting the model with the lowest BIC value). The optimal model (model type VVV, unconstrained model), found evidence for eight clusters, which corresponded moderately well with the 10 clusters described above. The adjusted Rand Index, a measure of the similarity of different clustering solutions between this partitioning and the *k* = 10 solution for the PAM, was 0.417 (0 means no agreement in assignments, 1 means identical assignments). When these clusters were merged using the demp method, however, the optimal number of clusters was found to be 2, corresponding again to high fundamental frequency elements versus all other types. The results of the GSI analysis of the PAM clustering and the BGM/demp analysis together suggest that there is only clear statistical evidence for partitioning zebra finch elements into these two broad categories, each of which encompasses a large range of structural variation.

**Figure 6:**
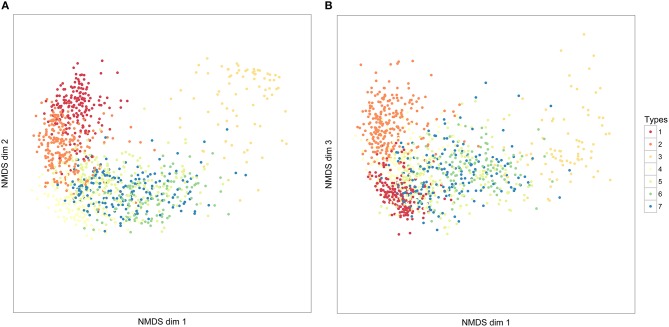
**The distribution of seven syllable clusters across three NMDS dimensions**. Each point represents an individual syllable. The closer syllables are to each other in these dimensions, the more similar they have been judged in similarity, according to our DTW comparison of syllables. The values in NMDS dimensions are not meaningful, and following convention, axes have not been labeled. **(A)** NMDS dimensions 1 and 2; **(B)** NMDS dimensions 2 and 3.

The global silhouette index of the PAM cluster analysis of syllables showed a clear peak at *k* = 7, although the low silhouette values suggested only a moderate tendency for syllables to form clusters (Figure 4). Examples of these clusters are shown in Figure 2C (See Table 3 for descriptions, Table 5 for summary acoustic parameters, for an illustration of how these clusters differ in the first three dimensions of an NMDS ordination of the DTW dissimilarities, see Figure 6). Three of the categories (1–3) corresponded to single elements that also occurred as “stand-alone” syllables, while the remaining four were compound syllables of varying complexity.

**Table 3:** **Description of syllable types identified by cluster analysis**.

**Type**	**Description**
1	Flat notes.
2	Slide notes.
3	High-pitch syllables.
4	Low frequency compound syllables—beginning with a flat or upsweep element and ending with a slide element.
5	High frequency compound syllables—typically with a high element toward the beginning, and typically declining in fundamental frequency; shorter than 7.
6	Complex compound syllable—with a high element in the middle or toward the end.
7	Flat-end compound syllable—typically containing a prominent flat element toward the end.

**Table 4:** **Summary statistic for acoustic parameters of the 10 element types identified by cluster analysis**.

**Type**	**Duration (ms)**	**Mean frequency (Hz)**	**Fundamental frequency (Hz)**					**Mean fundamental frequency change**	**Mean harmonicity**
			**Mean**	**Maximum**	**Minimum**	**Start**	**End**		
1	90.07 (42.39)	3454.32 (579.42)	642.7 (160.81)	736.98 (245.96)	527.1 (139.54)	**657.29 (223.82)**	**582.13 (198.5)**	**0.49 (0.03)**	0.89 (0.05)
2	43.83 (25.98)	4052.51 (672.11)	1126.38 (392.81)	1250.93 (433.19)	959.82 (376.06)	**1131.06 (426.42)**	**1040.1 (407.03)**	**0.48 (0.05)**	0.83 (0.07)
3	50 (25.69)	4143.06 (640.57)	916.71 (331.35)	1107.33 (428.01)	734.96 (290.13)	**953.35 (402.68)**	**834.55 (332.34)**	**0.48 (0.05)**	0.62 (0.05)
4	46.68 (19.84)	4140.88 (566.65)	804.68 (306.75)	1328.28 (807.36)	518.42 (185.8)	**1255.99 (796.18)**	**555.46 (207.12)**	**0.35 (0.06)**	0.68 (0.06)
5	50.73 (25.39)	3208.25 (543.44)	657.73 (199.59)	936.47 (435.64)	481.72 (153.33)	**883.53 (425.78)**	**517.18 (181.3)**	**0.41 (0.05)**	0.75 (0.07)
6	37.03 (23.75)	4526.24 (786.2)	**3067.91 (1279.76)**	3773.82 (1682.59)	2317.54 (1097.05)	3310.01 (1562.21)	2600.81 (1368.44)	0.44 (0.08)	0.74 (0.08)
7	24.61 (14.94)	3794.69 (1158.93)	**3588.44 (1402.63)**	4584.01 (1616.71)	2423.02 (1323.48)	4375.75 (1624.45)	2454.06 (1331.06)	0.27 (0.08)	0.91 (0.08)
8	26.73 (17.93)	4910.55 (1090.15)	**4779.82 (1234.35)**	5243.09 (1417.58)	4277.04 (1216.97)	4676.44 (1306.16)	4706.8 (1468.93)	0.51 (0.08)	**0.98 (0.04)**
9	24.49 (11.36)	1577.36 (501.5)	1328.38 (485.44)	1582.19 (510.41)	1030.61 (480.18)	1293.34 (537.97)	1280.43 (526.66)	0.50 (0.18)	**0.93 (0.08)**
10	19.52 (8.59)	3483.94 (946.63)	1139.02 (779.34)	1501.89 (1167.17)	791.48 (385.87)	862.07 (437.26)	1424.67 (1169.24)	**0.69 (0.1)**	0.69 (0.09)

Values show means (sd). Distinctive features of particular clusters are highlighted in bold.

**Table 5:** **Summary statistic for acoustic parameters of the seven syllable types identified by cluster analysis**.

**Type**	**Duration (ms)**	**Mean frequency (Hz)**	**Fundamental frequency (Hz)**					**Mean fundamental frequency change**	**Mean harmonicity**
			**Mean**	**Maximum**	**Minimum**	**Start**	**End**		
1	98.62 (43.23)	3546.11 (667.91)	775.61 (710.15)	1016.93 (1101.8)	578.59 (577.75)	**812.43 (781.33)**	**669.35 (626.83)**	**0.48 (0.04)**	0.87 (0.08)
2	64.74 (28.87)	3792.47 (615.94)	856.19 (723.03)	1505.21 (1416.1)	531.65 (576.7)	**1207.72 (957.92)**	**624.97 (677.51)**	**0.40 (0.06)**	0.73 (0.08)
3	58.92 (46.69)	4494.4 (1017.94)	**3634.51 (1936.38)**	**4571.17 (2342.15)**	**2757.68 (1801.63)**	**3852.94 (2085.22)**	**3285.86 (1995.39)**	0.45 (0.08)	0.91 (0.1)
4	129.22 (67.11)	3813.67 (568.4)	990.06 (659)	2046.67 (1651.4)	511.91 (506.13)	1185.21 (782.13)	825.88 (613.63)	0.44 (0.05)	**0.72 (0.08)**
5	107.59 (47.35)	3802.93 (587.2)	1294.98 (788.53)	**2604.5 (1922.33)**	583.96 (423.63)	1474.88 (822.61)	1088.29 (760.28)	0.44 (0.05)	0.8 (0.07)
6	188.24 (70.93)	3997.82 (506.29)	1866.82 (739.39)	**5252.54 (1886.98)**	526.56 (239.7)	2057.85 (935.45)	1622.94 (671.54)	0.45 (0.04)	0.76 (0.08)
7	135.49 (60.43)	3871.74 (619.93)	1443.52 (873.21)	3210.73 (2134.03)	594.42 (488.65)	1642.46 (972.69)	1237.11 (836.47)	0.44 (0.05)	0.78 (0.07)

Values show means (sd). Distinctive features of particular clusters are highlighted where appropriate in bold.

We also applied the BGM clustering algorithm to the syllable data, using 12 NMDS dimensions that accounted for 89% of the variation in the dissimilarity matrix. The optimal model found support for eight clusters, while demp, reduced this to seven clusters. These seven clusters corresponded moderately closely to the seven clusters found by PAM (adjusted Rand index: 0.513). In particular, the three single note syllable types (1–3) and type 7 each corresponded closely to a unique BGM cluster, while PAM type 4 was divided between BGM types 5 and 6.

### Song composition and syntax

Zebra finch motifs might be constructed simply by sampling syllables or elements at random from the population. We investigated whether there was any evidence that zebra finches deviate from this simple process.

**Figure 7:**
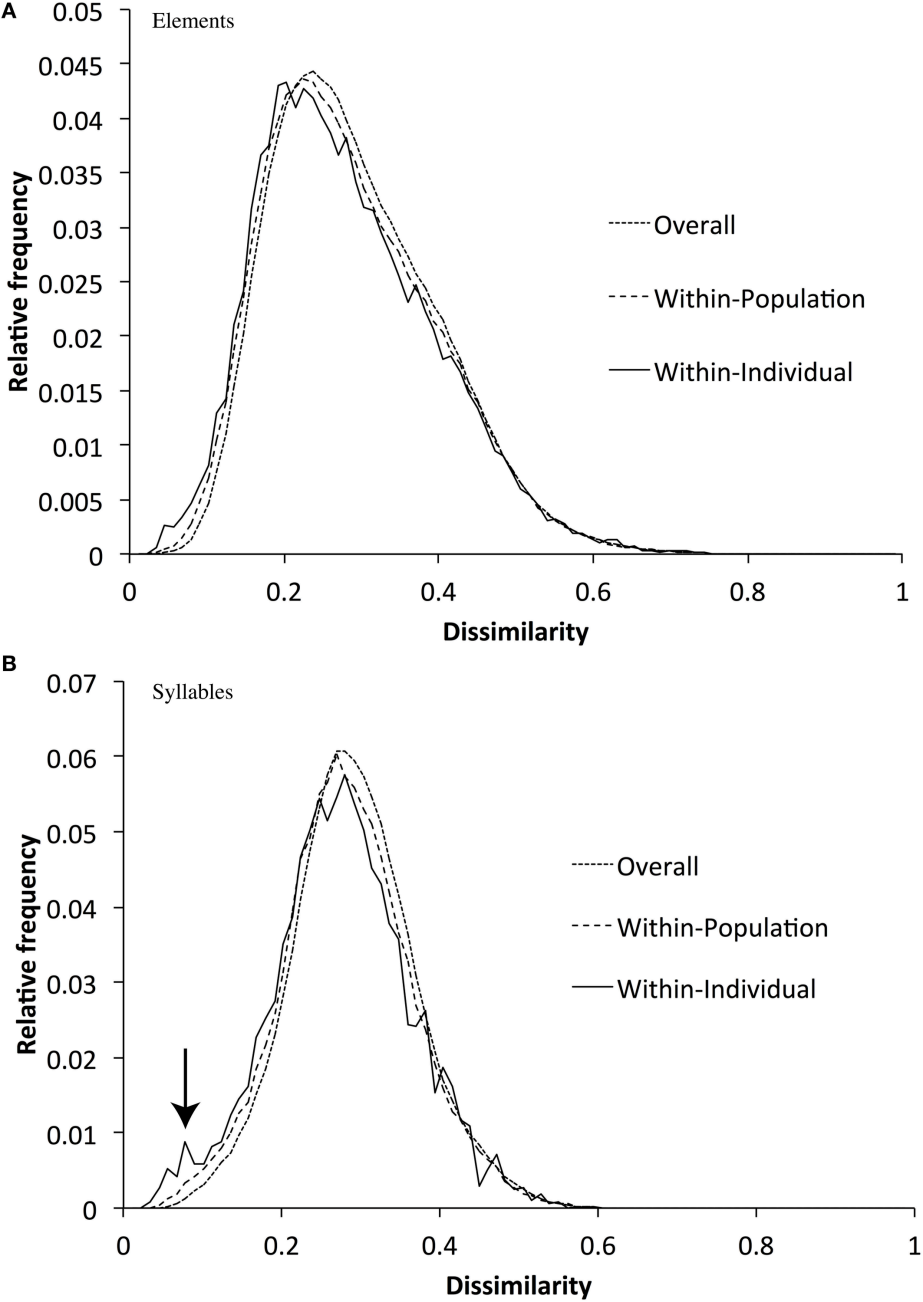
**Distribution of DTW dissimilarities over the whole sample, within populations, and within individuals for elements (A) and syllables (B)** (Within-population dissimilarity scores also include the within-individual scores, and the whole-sample scores include within-population scores). The figure shows that within-population dissimilarities are slightly lower (visible as a left-ward shift of the curve) than dissimilarities within the whole sample, and that within-individual dissimilarities are similar to within-population dissimilarities.

For example, each zebra finch might attempt to develop a motif that contains all of the different broad syllable types (if, for example, different syllable types served different communicative roles). If so, we would predict that motifs would contain more syllable types than expected by chance. We tested this hypothesis by randomly permuting elements or syllables between motifs (but within populations), and measuring whether the number of types found per motif was greater in the empirical data-sets than in the randomly sampled data-set. In fact, we found that zebra finch motifs had slightly fewer element types (mean = 6.39) than expected by chance (expected = 6.56, *p* < 0.01), and almost exactly the same number of syllables (mean = 3.92) as expected by chance (expected = 3.96, *p* > 0.2). We also found little evidence that elements or syllables varied less (or more) within songs than within populations (Figure 7)—although there was a trend for motifs to contain higher proportions of very similar syllables (a small peak at the far left of the graph). This trend is likely caused by a small number of individuals repeating part of the motif.

**Figure 8:**
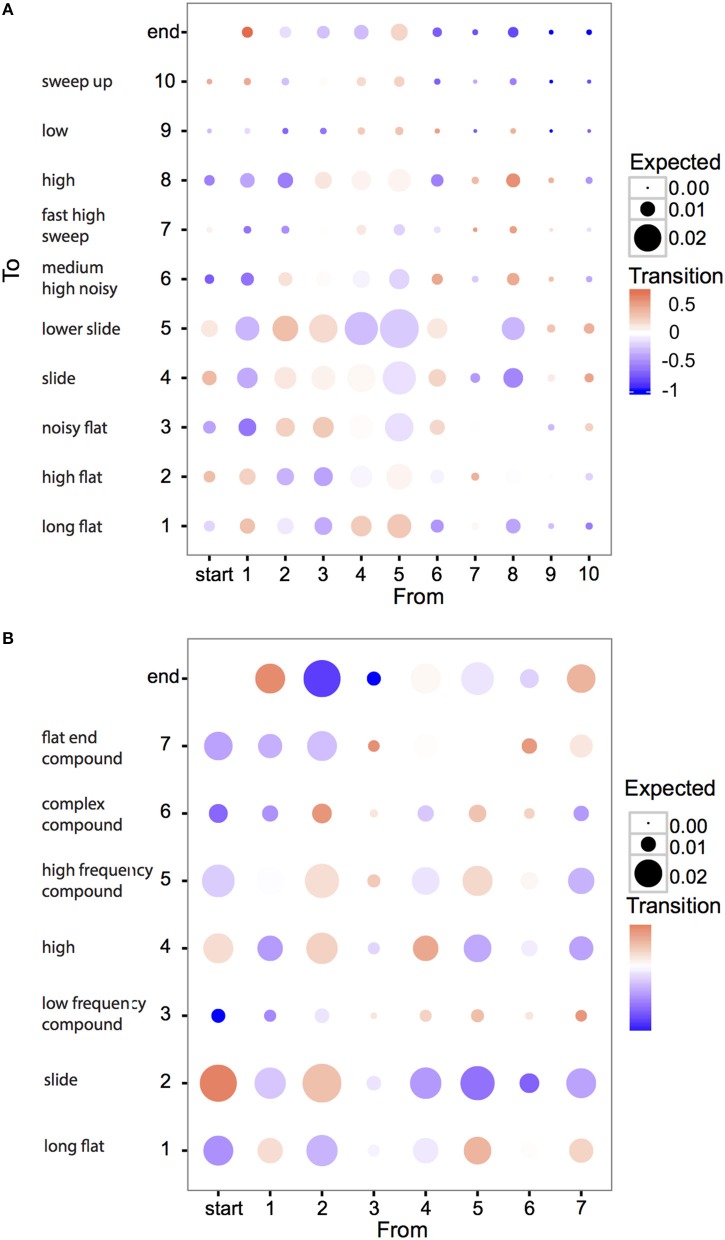
**Biases in transitions between zebra finch elements (A) and syllables (B)**. Each circle represents the transition between two element or syllable types. Numbers on the axes correspond to element and syllable types as described in the text and Figure 2 and matching short descriptions of each type correspond to Tables 2, 3. For example, the bottom right corresponds to the case where element type 10 is followed by type 1. Colors show the degree to which transitions occur more or less frequently than expected by chance, calculated as: (p_obs_ − p_exp_)/max(p_obs_, p_exp_) where p_obs_ and p_exp_ are observed and expected probabilities. If p_obs_ > p_exp_, the color scale is red; if p_obs_ < p_exp_, the scale is blue; if p_obs_ = p_exp_, the color is white. The size of the circle corresponds to the expected probability of the transitions by chance (since some sound units occur more frequently than others, the chance they occur in a transition is also higher). Thus, large, brightly colored circles represent large deviations from expected probabilities for transitions between sound units that have large sample sizes. Most circles are not brightly colored, suggesting a low deviance from expected frequencies. However, the exceptions to this that represent stronger deviations from expected frequencies, might explain why the syntactical structure of the songs, as measured by Markov-chain redundancy, is slightly higher than expected by chance.

An alternative form of structure in motif construction is syntactical dependency: does the appearance of an element or syllable depend on the preceding (or following) unit? To explore this, we first examined whether there were obvious examples of such dependencies in individual element or syllable types (Figure 8, brighter colors indicate higher (red) or lower (blue) probability of transition between sound units than expected). We found that most inter-element or inter-syllable transitions occurred about as frequently as expected, based on element and syllable frequencies. But there were several exceptions to this rule: flat notes (E1, S1) tended to occur at the end of motifs, but not at the beginning. Conversely slide notes (E4, E5, but especially S2) were concentrated at the beginning of motifs, and were absent from the end. The accumulation of these units at the beginning and end of motifs confirms previous observations based on subjective categories, and has been hypothesized to be the result of the incorporation of call notes into songs (Zann, 1993a): both slide and flat elements resemble different zebra finch calls.

High frequency notes (E8, S3) and low bandwidth elements (E6–E10) were rare at either the start or the end of motifs. For syllables, all seven types were followed by a syllable of the same type more often than expected. One hypothesis to explain this is that identical syllables are sometimes repeated within motifs, but this happens only rarely. An alternative explanation is that the tendency to repeat general syllable *types* might reflect a bias imposed by production effort: to switch from one type of syllable to another might reflect a larger change in the position of the vocal organs than producing another syllable of the same general type. Related to this result is a second: the tendency for high syllable types (S3), and compound syllables ending with a high element (S6), to be followed by a compound syllable beginning with a high element (S7). Finally, several transitions occurred at higher or lower probabilities than expected, without any obvious explanation: notably the tendency of slide syllables (S2) to be followed by a particular type of compound syllable (S6).

Of course, one might expect that some transitions might occur more or less frequently than expected as a result of sampling error, and the question arises whether the frequent transitions described above reflect genuine syntactical constraints. One way to investigate this is to measure the first-order Markov entropy of element and syllables—an estimate of the predictability of sequences. We measured first-order entropy (with a correction for finite sample sizes), using the approach developed by Lachlan et al. (2013). This method clusters elements or syllables partly on the basis of what partition will maximize syntactical structure. We rescaled the results in the form of redundancy, where 0 represents no syntactical structure and 1 represents maximum syntactical structure. For finite sample sizes, it is to be expected that even random data-sets will have redundancy estimates >0 using this method (due to the clustering algorithm searching for partitions with maximum redundancy); to account for this we also applied our method to randomly generated dissimilarity matrices (using the standard deviations of 50 NMDS dimensions taken from the empirical data as a basis). Confidence limits for redundancy estimates were established by bootstrapping (see Lachlan et al., 2013 for details).

**Figure 9:**
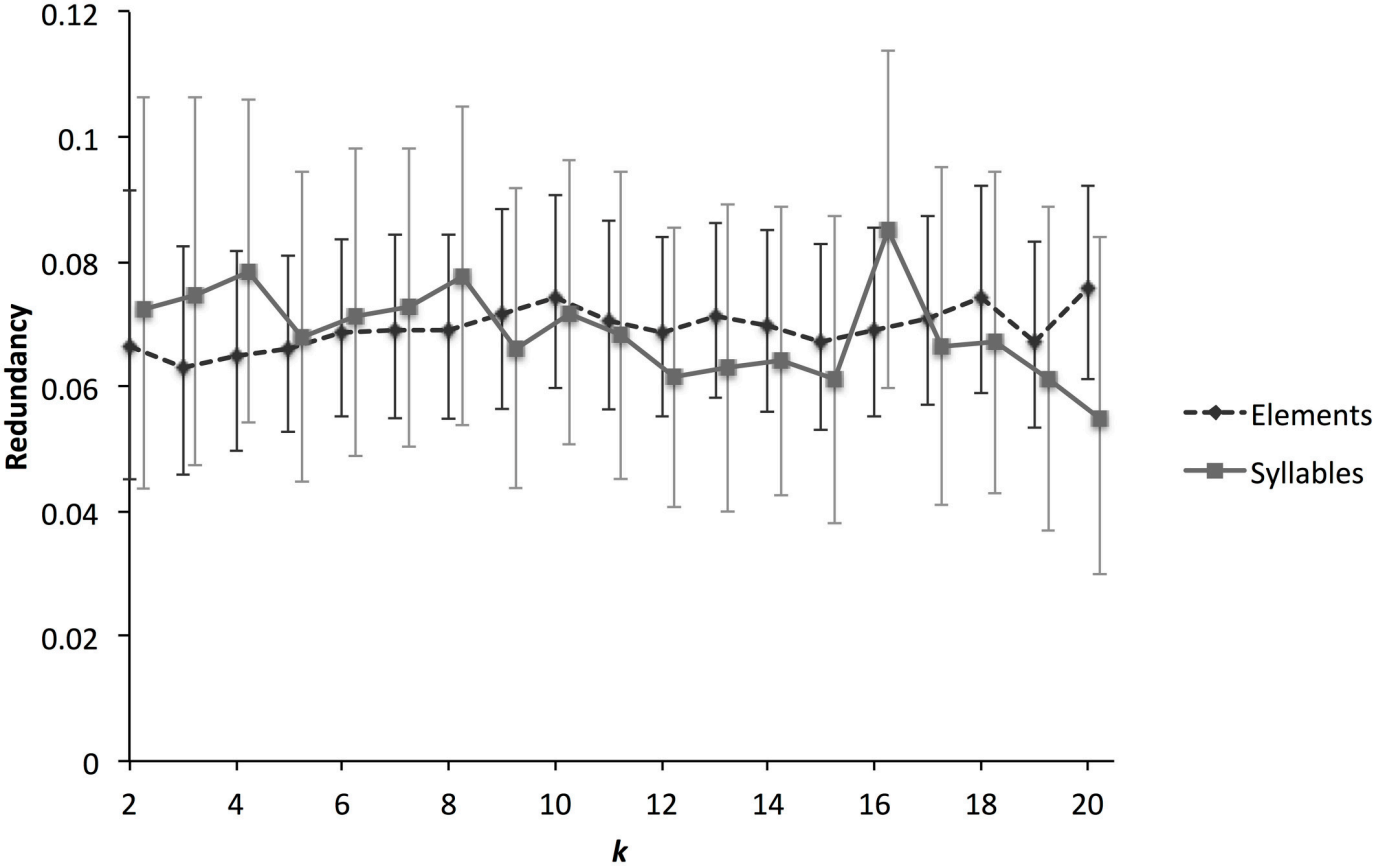
**Syntactical structure for zebra finch elements and syllables**. The graph shows how a measure of syntactical structure, estimated Markov chain redundancy, varies with different numbers of syntactic clusters. This clustering algorithm partitions data according to acoustic structure and maximizes estimated redundancy. Because of this, randomly simulated data is predicted to have redundancy greater than 0. We therefore simulated data-sets (three times each for elements and syllables), and estimated redundancy in the simulated data-set. The estimated empirical redundancy scores were then corrected by subtracting the simulated scores. Higher levels of redundancy correspond to greater syntactical structure. Values greater than 0 represent more structure than expected by chance. Error bars represent the 99% Conficence Interval, estimated by bootstrapping.

This analysis finds low levels of syntactical structure for both elements and syllables (Figure 9), although, in both cases, levels are significantly higher than for the randomly generated data. This means that there is some predictability in how elements and syllables are combined, but in general sequences are only slightly more predictable than random ones. There was no evidence that redundancy was higher for syllables than for elements. A contrast can be drawn between redundancy estimates for zebra finches and those made for several populations of chaffinches (*F. coelebs*) (Lachlan et al., 2013). While zebra finch redundancy was lower than 0.1, European chaffinch populations had entropy values greater than 0.5. In fact, zebra finch syntactical structure is similar and even slightly lower than that of Gran Canaria chaffinch populations, who appear to have lost most of their syntactical structure.

In summary, there appear to be very few constraints or biases influencing how zebra finches construct motifs from their constituent units. Zebra finch motifs seem, in general, to be random sequences of syllables with few exceptions to this rule.

### Population divergence in acoustic song structure

Within population element and syllable dissimilarities were lower than dissimilarities calculated over all populations combined (Figure 7). We tested the significance of this using a multi-response permutation procedure (MRPP, McCune and Grace, 2002), in which elements or syllables are permuted between populations. The result of this analysis (elements: δ = 0.2841, m_δ_ = 0.2887, effect size *A* = 0.0159, *p* < 0.001; syllables: δ = 0.2818, m_δ_ = 0.2879, *A* = 0.0213, *p* < 0.001) suggests that average dissimilarities between elements or between syllables are lower within than between populations, and that therefore populations have diverged in acoustic element and syllable structure, but that the effect size is very small. This divergence could, however, be simply due to variation between populations in the relative frequency of use of the different element or syllable types (i.e., in how often an element or syllable occurs in one population compared to another population rather than in how different element types are between population). Alternatively, the acoustic structure of elements or syllables within types could vary between populations. We investigated these possibilities separately.

**Figure 10:**
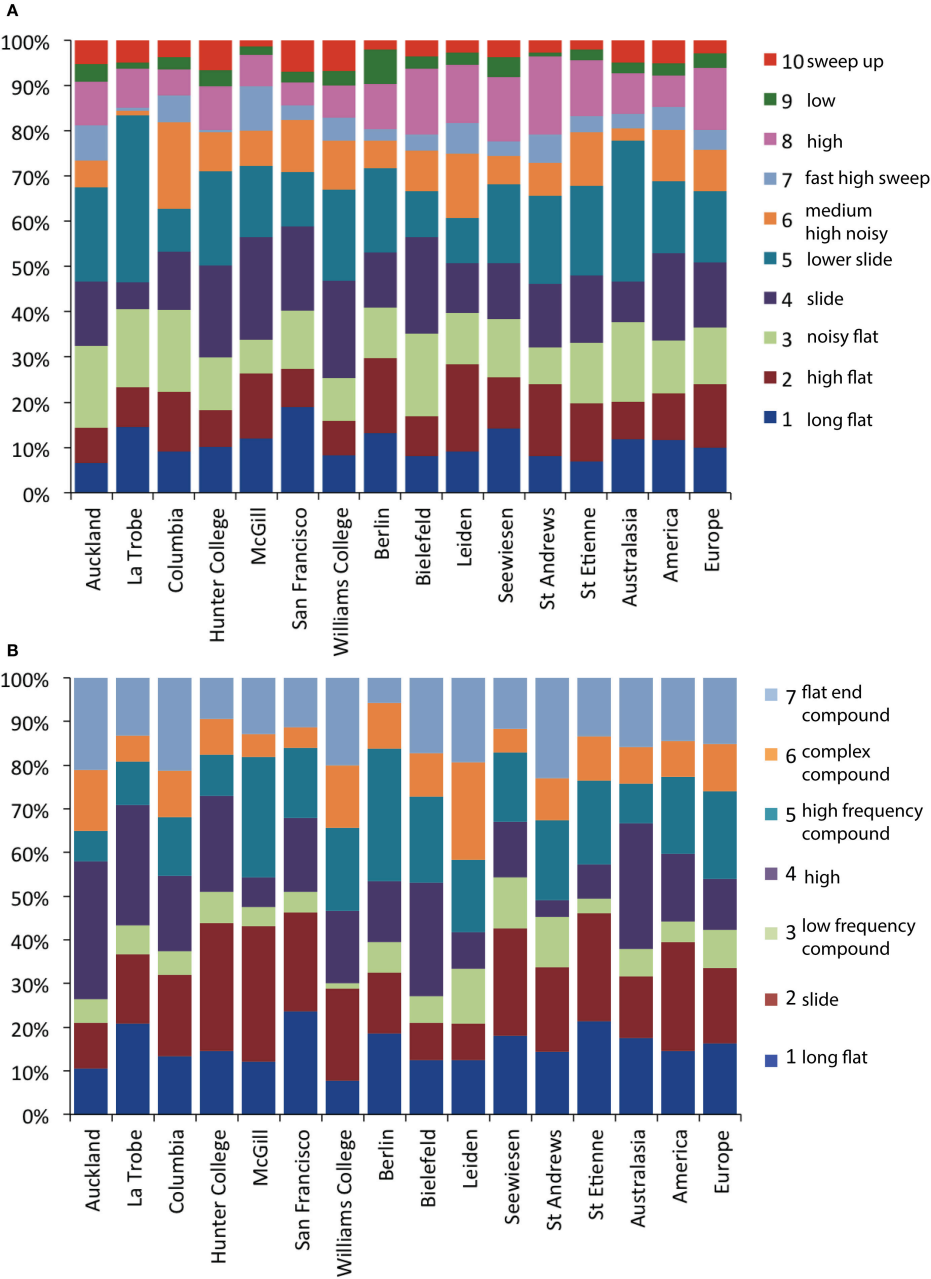
**The proportion of members of element (A) and syllable (B) cluster types in each of the 13 populations and each of the three continents**. Color labels refer to the cluster types described in the text and Figure 2 and matching short descriptions of each type correspond to Tables 2, 3.

Both elements (χ^2^ = 409.6, *df* = 108, permuted *p* < 0.0001) and syllables (χ^2^ = 184.3, *df* = 72, permuted *p* < 0.0001) were used at different frequencies of occurrence in different populations (Figure 10). However, it is also notable that all element and all syllable types were present in all populations. Cramér's V, a measure of association in contingency tables, was also low (elements: ϕ_C_ = 0.128, syllables: ϕ_C_ = 0.161), suggesting that although there were significant differences between populations, the corresponding effect size was low.

We examined the degree to which elements and syllable clusters from the 13 populations diverged, also using an MRPP permutation approach. We first analyzed population differentiation for each of the element and syllable types. All 10 element types showed significant levels of differentiation between populations, as did 6 of the 7 syllable types (Table 6); high frequency note syllables were the only type not to show significant differentiation. But effect sizes were low in both cases—especially with elements (Maximum effect size, A: 0.091 for low notes; 0.051 for complex syllables with a high frequency element toward the end).

**Table 6:** **Shows the results of MRPP analyses of population divergence in element (A) and syllable (B) structure for each of the element or syllable types**.

**Type**	**δ**	***m*_δ_**	***p***	***A***
**(A)**				
1	0.146	0.153	<0.0001	0.0455
2	0.179	0.185	<0.0001	0.0356
3	0.183	0.189	<0.0001	0.0292
4	0.181	0.187	<0.0001	0.0335
5	0.178	0.182	<0.0001	0.0262
6	0.219	0.228	<0.0001	0.0422
7	0.241	0.249	0.0027	0.0300
8	0.182	0.188	<0.0001	0.0330
9	0.300	0.331	0.0002	0.0912
10	0.273	0.282	0.0012	0.0333
Overall	0.190	0.197	<0.0001	0.0365
**(B)**				
1	0.149	0.156	<0.0001	0.0475
2	0.182	0.192	<0.0001	0.0506
3	0.241	0.244	0.1527	0.0139
4	0.244	0.253	<0.0001	0.0335
5	0.245	0.253	<0.0001	0.0346
6	0.269	0.284	<0.0001	0.0509
7	0.254	0.265	<0.0001	0.0433
Overall	0.220	0.230	<0.0001	0.0404

δ is the observed test statistic; m_δ_ the expected test statistic, p the p-value associated with the difference between the two, and A an effect size measure. A significantly smaller δ than m_δ_ indicates population divergence (higher between- than within-population variation). The Overall row refers to results obtained when permuting all elements or syllables, but only within their type category.

**Figure 11:**
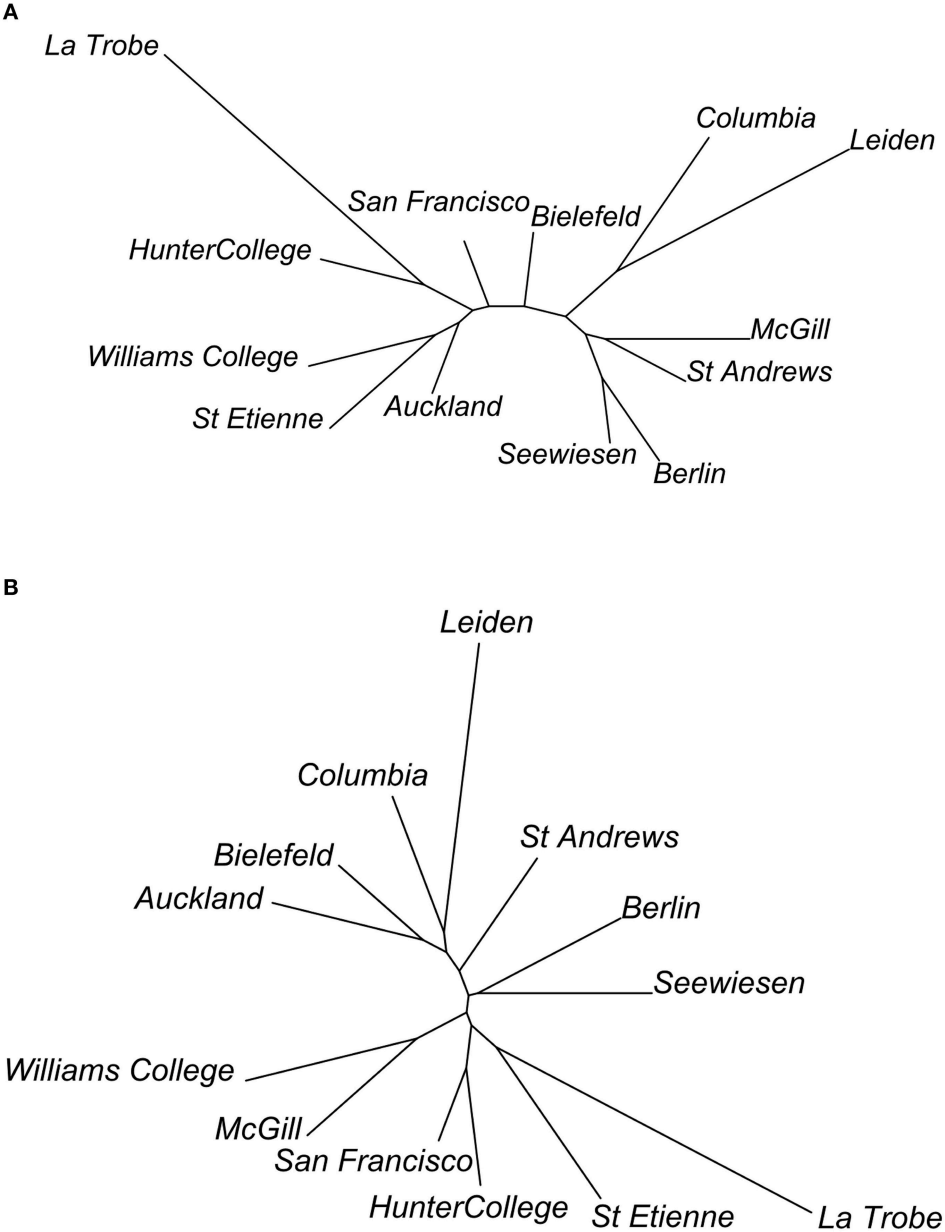
**Dendrograms of relationships between populations, based on (A) element and (B) syllable dissimilarity**. Dendrograms are calculated as neighbor-joining trees, using the dissimilarities between population spatial medians.

We were able to examine population differentiation in a different way by finding the spatial median of the motifs of each population, then measuring the DTW motif similarity between them, and finally constructing neighbor-joining dendrograms (Figure 11). In the case of syllables, but not for the elements, European and American populations tended to cluster together (except for the Bielefeld population). For both syllables and elements, La Trobe, the wild-caught Australian sample, appeared as an outlier. Based on this, we repeated the analyses described above, but grouping individuals by continent rather than by population.

Overall, divergence between continents was lower than between populations (elements: observed δ = 0.2869, expected m_δ_ = 0.2887, effect size *A* = 0.0060, *p* < 0.001; syllables: δ = 0.2858, m_δ_ = 0.2879, *A* = 0.0073, *p* < 0.001 as compared to *A* = 0.0159 and *A* = 0.0213 respectively for element and syllable population divergence). Elements (χ^2^ = 161.3, *df* = 18, permuted *p* < 0.001) and syllables (χ^2^ = 54.6, *df* = 12, permuted *p* < 0.001) were used at different frequencies of occurrence in different continents (Figure 10). But Cramér's V values were similar to population comparisons (elements: ϕ_C_ = 0.170, syllables: ϕ_C_ = 0.140), suggesting low effect sizes again. Finally, 7 of the 10 element types and 6 of the 7 syllable types differed in acoustic structure between continents (Table 7). As with the comparison between colonies, effect sizes were small (maximum A: 0.020 for flat elements, and 0.021 for flat-note syllables).

**Table 7:** **Shows the results of MRPP analyses of continent divergence in element (A) and syllable (B) structure for each of the element or syllable types**.

**Type**	**δ**	***m*_δ_**	***p***	***A***
**(A)**				
1	0.149	0.153	<0.0001	0.0207
2	0.183	0.185	<0.0001	0.0122
3	0.187	0.189	<0.0001	0.0092
4	0.185	0.187	<0.0001	0.0090
5	0.181	0.182	<0.0001	0.0071
6	0.228	0.228	0.1538	0.0013
7	0.251	0.251	0.7661	−0.0027
8	0.188	0.188	0.0894	0.0025
9	0.326	0.331	0.0451	0.0136
10	0.278	0.282	0.0064	0.0132
**(B)**				
1	0.153	0.156	<0.0001	0.0213
2	0.189	0.192	<0.0001	0.0131
3	0.247	0.247	0.3987	0.0005
4	0.250	0.253	<0.0001	0.0119
5	0.251	0.253	<0.0001	0.0092
6	0.281	0.284	0.0009	0.0096
7	0.262	0.265	<0.0001	0.0133

δ is the observed test statistic; m_δ_ the expected test statistic, p the p-value associated with the difference between the two, and A an effect size measure.

## Discussion

Beyond the division of elements into high and low pitch categories, our analysis found only weak evidence for species-wide clustering in zebra finch elements or syllables. Clustering tendency was weak, and the results of different clustering algorithms were only broadly similar. Some evidence for clustering was found for syllables, which were divided into potentially biologically relevant categories, identifying, notably, “slide” and “flat” syllable categories that are likely adapted call notes. Given the high variability of zebra finch song motifs, and the widespread sources of songs for this study, this is itself notable. The results of the clustering analysis are reflected in our analysis of motif structure and syntax: while there is only weak syntactical structure overall, certain transitions were highlighted as significantly different from expected probabilities. Notably, again, these often involved “slide” and “flat” syllables, concentrated at either end of the motif. These results indicate that relatively isolated laboratory populations, show low levels of song divergence between populations, similar to tendencies in wild zebra finch populations.

We interpret these results to suggest that while there are some underlying constraints on the acoustic structure of zebra finch elements or syllables, these provide considerable latitude for variation. Within broad species-specific limits, zebra finches might learn almost any element or syllable structure, and songs consist of an almost random sequence of elements and syllables. There are only a few exceptions to this permissive system, which restrict within-species song variation: the division of notes into high and low pitch categories; the clear slide and flat note categories, and their restriction to either end of the song motifs; and the tendency of syllables of the same broad type to repeat themselves within the motif.

Likely sources for the biases that underlie these exceptions can be identified in most cases. Zebra finches typically produce sound through exhalation, but high frequency notes are thought to be often, although not always, the result of inhalation rather than exhalation (Goller and Daley, 2001). This may also explain their tendency to be produced around the middle of the song rather than at the beginning or the end, as they may enable the birds to keep singing where they would otherwise run out of breath. This alternative, and possibly more limited, form of phonation may also explain why high-note syllables did not significantly differ in acoustic structure between populations. Slide and flat notes are very similar in acoustic structure to communication calls, stack and distance calls, that appear to have been incorporated into songs (Price, 1979; Zann, 1993a). This may also explain their tendency to appear at the beginning and ends of motifs. Repetition of syllable categories might possibly be the consequence of a bias toward easier-to-produce motifs, that require less dramatic movements of the vocal organs from one syllable to the next, mirroring well-understood limits to vocal production in other species (cf Podos, 1997). Recent evidence also suggests that young zebra finch males raised without exposure to male song also have perceptual biases in favor of more common element types (stacks and slides) compared to rarer (high frequency and noisy) types (ter Haar et al., 2014). However, it remains an open question whether this translates into perceptual correlates of individual element categories. These perceptual biases affect song imitation, although learning by experience could at least partially override the initial perceptual biases (ter Haar et al., 2014). The overriding effect of experience allows imitation beyond these biases, which may explain why there are only few constraints found at the species level.

Zebra finch songs are culturally transmitted, and therefore the sources of weak constraints on song variation discussed above are likely to act as “selection pressures” directing cultural evolution. And similar to how small selective advantages can shape genetic evolution, even very weak underlying biases influencing song learning can result in strong changes in patterns in cultural traits, such as birdsong (Smith, 2011). It might be that calls are only rarely incorporated into songs during development, or that repeated syllable types are only slightly easier to produce, but cultural evolution may have amplified these biases into the quite clear patterns we observed.

Previous research on population differentiation and song note classification of laboratory populations (Sturdy et al., 1999) was based on visual inspections of the spectrograms and a relatively low sample size, which was appropriate for the aim of that study (describing harmonic structure and note order stereotypy). By using a computational measure of similarity, and larger sample sizes, both in terms of subjects per population and the number of populations, we were able to employ statistical measures of clustering and a wider range of tests of divergence between populations. Although we only found weak clustering tendencies, it is notable that there was some overlap with earlier descriptions of syllable or element categories (Zann, 1993b; Sturdy et al., 1999) especially for slide notes (E4, S2), flat notes (E1, E2, S1) and high frequency notes E8, S3). Our study also complements another recent quantitative analysis of zebra finch vocalizations (Elie and Theunissen, 2015), which uses a quite different suite of methods to measure vocal dissimilarity in order to understand acoustic differences between zebra finch vocalizations produced in different contexts (i.e., different call types). The diversity of computational methods available raises the possibility that our analysis may be dependent on the particular methods that we applied. It is clear that there is no one computational comparison method for animal sounds that has unimpeachable validity, and we would urge further integration of such methods with behavioral studies of song perception.

We measured statistically significant divergence in the acoustic structure of elements and syllables between populations, but the differentiation was very weak, and we doubt whether this result would have any communicative significance. It seems unlikely that zebra finches would prefer an unfamiliar song from their own population over a song from another population, as has been found in many other songbird species. One reason for this prediction is that we did not find any cases of a broad syllable or element type being entirely absent from a population, even with the relatively small sample sizes that we used. In addition, although there was some between-population variation in the acoustic structure within element or syllable types, this variation was again not large.

This result supports a previous study that found little divergence in the acoustic structure of songs between wild zebra finch populations (Zann, 1993b). In that study, the reasonable explanation was provided that populations may not be isolated from one another—frequent interchange of individuals between populations can prevent divergence. That explanation may to some extent also apply to the populations in our study, as some labs may have exchanged birds. However, while there was also some influx to the laboratory colonies from breeders' stock, this did not occur frequently or in all colonies, and certainly did not occur between all colonies (such as between continents). The fact that laboratory populations on different continents show some genetic differentiation (Forstmeier et al., 2007) suggest the effect of mixing may be limited. Another explanation for the weak levels of differentiation is thus required.

One possibility is that there has not been sufficient time for cultural or genetic divergence to occur since zebra finches were first domesticated ~150 years ago. Given our knowledge about the precision of zebra finch song learning, the results of our simulations of cultural evolution (Figure 1), and the findings of genetic differentiation between laboratory colonies (Forstmeier et al., 2007), it seems unlikely that there has been insufficient time for cultural divergence. But there may well not have been enough time for divergence in the genetic underpinnings of song learning. This might explain the difference between the zebra finch situation, showing marginal differences between the wild population and domestic ones, and that of the Bengalese finch that has had about 260 years history of domestication and that shows considerable divergence of the domestic strains' songs from the ancestral songs (Honda and Okanoya, 1999). However, it is unknown to what extent different domesticated populations of Bengalese finches differ from each other. Bengalese finches have been domesticated over a longer time period and wild and domestic populations might thus show more genetic differentiation than the differentiation existing among zebra finch populations. On the other hand enough time has occurred for significant genetic divergence to arise at some loci between zebra finch population (Forstmeier et al., 2007). It may be that, given further time, evolution in the constraints underlying learning would lead to divergence in the elements and syllables produced in different populations. Interestingly, the divergence between wild and domesticated population was slightly stronger than among domesticated populations, suggesting that such a process might be beginning.

A more probable explanation for the minimal cultural divergence between populations is that high error rates, combined with general constraints on the acoustic structure of syllables and elements, lead to homogenization between populations. In order for cultural evolution to lead to stable change, such as the divergence of two isolated populations, cultural transmission must reach a threshold of precision (Figure 1). Our results may show what happens when that threshold is not reached. Previous experimental studies of song learning in zebra finches have found that tutees on average learn ~50% of their elements from primary tutors (Jones et al., 1996; Houx and ten Cate, 1999; Holveck et al., 2008). Although some birds copy songs almost perfectly, others copy very little from tutors (Bolhuis et al., 2000; Tchernichovski and Mitra, 2002). The development of the remaining 50% of elements is less well understood—they may be influenced by other songs in the population, such as those of group mates (Mann and Slater, 1995; Jones et al., 1996), even if the results do not closely resemble any one tutor element. But nevertheless, improvisation and innovation clearly play a large role in zebra finch song development, and this appears to prevent the formation of local traditions.

Our results support the contention that cultural transmission does not always lead to cultural evolution. In a recent experimental study of cultural transmission over several generations, it was shown that isolate zebra finch song quickly returned to species specific norms (Fehér et al., 2009), and this was suggested to be an example of cumulative cultural evolution. While this may be true to a degree, it also suggests the presence of constraints acting as selection pressures, driving the songs to the species wide characteristics. If such processes also lead to the homogenization of populations, it certainly limits the active role that culture can play in zebra finch song evolution.

## Author contributions

The study was conceived with major contributions from all authors, and all authors contributed to the writing of the manuscript. RL, CvH, and StH contributed equally to the study. RL designed the analytical approach, carried out bioacoustic measurement in Luscinia and statistical analysis in R. CvH and StH collected and collated sound recordings, and carried out bioacoustic measurement in Luscinia and performed statistical analyses in R.

## Funding

StH was supported by an LIBC grant to Clara C. Levelt and CtC.

### Conflict of interest statement

The authors declare that the research was conducted in the absence of any commercial or financial relationships that could be construed as a potential conflict of interest.
