# Supplementary material for: Zebra Finch Song Phonology and Syntactical Structure across Populations and Continents—A Computational Comparison
Source: Front Psychol. 2016 Jul 7;7:980. doi: 10.3389/fpsyg.2016.00980 (PMC4935685; doi:10.3389/fpsyg.2016.00980)
Supplement: Supplementary file 1 [file DataSheet1.docx]

**Appendix – Methods for Cultural Evolution Simulations**

We designed individual-based simulations to examine cultural divergence between two recently founded populations. The particular design of the simulation was intended to approximate the history and demographics of laboratory zebra finch population and the organization of zebra finch song and song learning.

In each simulation, we began with one large unstructured population consisting of 1000 individuals. We simulated cultural transmission in this population for a period of 10,000 years. At this point, we randomly selected two sets of 100 individuals from the population to found new populations (the simulated captive populations). We then continued the simulation for a further 200 years. During this period, we compared song structure between the populations every 10 years.

Each zebra finch had a 40% chance of dying each year, upon which it was immediately replaced. A replacement bird then learned its song by selecting syllables randomly from the population. Its song then did not change for the remainder of its life. Each bird’s song consisted of 10 syllables. Each syllable was characterized by two numbers (range 0-1), representing ‘acoustic variables’. At the beginning of each run of the simulation, members of the undifferentiated population were given pseudo-random syllable values (uniform distribution). Each time an individual learned a song, they selected each syllable randomly from the songs of adults in the population. If they had already learned that syllable in their song, they resampled the population (up to 10000 times) until they found a syllable they hadn’t already learned. If they still hadn’t found a novel syllable, they improvised one. Apart from this extremely rare scenario, there was a fixed probability of errors/improvisation in learning for each syllable learned. If a syllable was improvised, birds generated a new syllable type in the same way that founders were provided songs: new values were drawn from a pseudo-random uniform [0,1] distribution.

This model of cultural transmission imposed fixed limits [0,1] in two dimensions on syllable structure. These limits were intended to model unlearned constraints on what zebra finches can learn. Our model of errors/improvisations in learning assumed that when a new syllable was generated, there was no input at all from existing syllables (i.e. birds did not modify existing syllables). We are aware of no evidence to support or contradict this assumption at present for the proportion of syllables that cannot be traced to a particular tutor. Similarly, when birds learned a syllable, they learned it entirely precisely. This approximation is certainly not entirely true. A more complex, realistic model would likely assume that syllable learning was rarely entirely precise, but that even non-imitated syllables may be based at some level on a model. With our current knowledge, such a model would involve adding considerable undesirable complexity to the model without having any empirical basis to assign values for the additional parameters, and (in preliminary results) with rather little impact on the outcome of the simulations.

To analyze songs from the population, we first calculated a dissimilarity score between each pair of songs in the population at each time point sampled. Each syllable was characterized by a real-valued number between 0 and 1; the dissimilarity between two syllables was the absolute difference in these numbers. To calculate a dissimilarity score between two songs, for each syllable in song A, we found the most similar syllable in song B, and then averaged over the whole song. We repeated this for song B relative to song A and summed the two scores to create an overall symmetric dissimilarity matrix between all sampled songs.

To visually explore divergence between populations, we carried out a Principal Coordinates analysis of the dissimilarity matrix and plotted the first two dimensions (which explained between 80% and 90% of total variation in all cases).

To analyze differentiation between populations, we calculated the mean dissimilarity between songs within populations and the mean dissimilarity of songs between populations. We then calculated the Global Silhouette Index between the songs using these values. Higher values of the GSI (near 1) indicate that the populations were clearly differentiated. Values near 0 represent no significant differentiation.

The simulations were implemented in Java. Code is available from the authors.
